# A STEMI Complicated by Cardiogenic Shock Due to Simultaneous Acute Thrombosis of Two Coronary Vessels in the ‘Deadly Double Infarct Syndrome’: A Case Report and Discussion of Literature

**DOI:** 10.3390/jcm13247511

**Published:** 2024-12-10

**Authors:** Gianluca Guarnieri, Daniela Mele, Daniele Briguglia, Massimo Medda, Edoardo Conte, Antonio Bartorelli, Daniele Andreini

**Affiliations:** 1 Department of Biomedical and Clinical Sciences, University of Milan, 20122 Milan, Italy; daniele.andreini@unimi.it; 2 Division of University Cardiology, IRCCS Ospedale Galeazzi Sant’Ambrogio, 20157 Milan, Italy; danieladoc93@gmail.com (D.M.); daniele.briguglia@grupposandonato.it (D.B.); massimo.medda@grupposandonato.it (M.M.); edoardo.conte86@gmail.com (E.C.); antonio.bartorelli@grupposandonato.it (A.B.)

**Keywords:** STEMI, cardiogenic shock, dual culprit lesion

## Abstract

**Background**: ST-segment elevation myocardial infarction (STEMI) remains a leading cause of mortality worldwide, primarily caused by acute thrombosis over atherosclerotic plaques. Simultaneous acute thrombosis in two coronary arteries is an exceptionally rare event. This report highlights a unique case of STEMI associated with cardiogenic shock due to dual coronary artery thrombosis and provides insights from a literature review on this rare condition. **Methods**: We report the case of a 58-year-old male with a history of hypertension, type II diabetes, and heavy smoking, who presented with a two-day history of chest pain and cardiogenic shock. Diagnostic evaluation included an electrocardiogram showing ST-segment elevation in AVR and ischemia, along with echocardiography revealing severe left ventricular dysfunction (ejection fraction 20%). Emergency coronary angiography was performed to identify the underlying pathology. Additionally, a literature review was conducted to analyze the characteristics and outcomes of similar cases of dual coronary artery thrombosis. **Results**: Coronary angiography identified significant occlusions in the proximal circumflex branch and the left anterior descending artery (LAD), a combination rarely reported in the literature. Our review confirmed that dual thrombosis involving the LAD and right coronary artery (RCA) is the most frequently described presentation of this condition, while simultaneous CFX and LAD thrombosis is exceedingly rare. Most reported cases, including ours, were associated with cardiogenic shock, highlighting the severity of this clinical entity. Despite successful thrombus aspiration and stenting, our patient experienced severe complications, including infections, pleural effusions, and paralytic ileus, ultimately requiring evaluation for left ventricular assist device implantation. **Conclusions**: This case underscores the complexity and critical challenges of managing STEMI with cardiogenic shock due to simultaneous coronary thrombosis. The findings from our literature review suggest the need for heightened clinical awareness and tailored revascularization strategies. Further studies are warranted to optimize management approaches and improve outcomes in such rare and high-risk scenarios.

## 1. Introduction

The ST-segment elevation myocardial infarction (STEMI) remains the leading cause of death worldwide. In most cases, it is caused by an acute thrombosis over an atherosclerotic plaque, resulting in downstream vessel occlusion and subsequent death of cardiomyocytes. Here, we present an extremely rare case where acute plaque thrombosis occurs simultaneously in thew left anterior descending artery (LAD) and in the circumflex artery, leading to a clinical picture of STEMI associated with unstable presentation of cardiogenic shock.

## 2. Case Presentation

A 58-year-old male with a medical history of hypertension, type II diabetes mellitus, and a current heavy smoker (approximately three packs per day) with pulmonary fibrosis and without a previous history of coronary artery disease, hypercoagulable state, or substance misuses was admitted to the emergency department following a two-day history of chest pain. On arrival, he was in cardiogenic shock, with cold and mottled skin and a systolic blood pressure of 70 mmHg, with the emogas analysis showing metabolic acidosis with a slight increase in snerum lactates (lactates 4 mmol/L).

**Figure 1:**
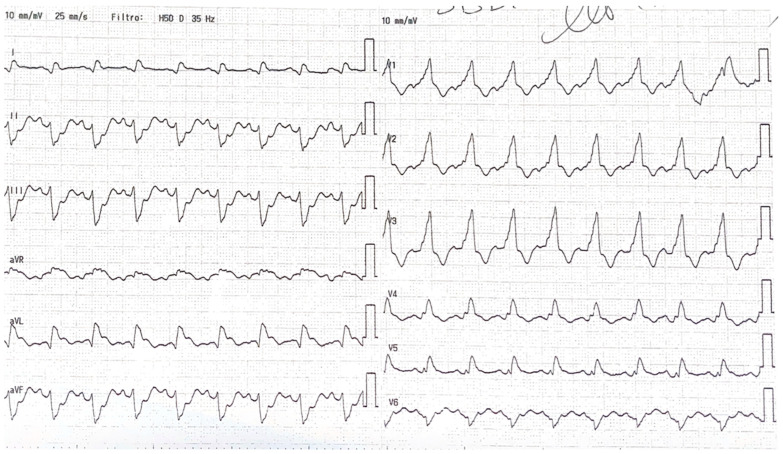
the ECG showing ST-elevation in aVR, aVL and DI and right bundle branch block.

The initial electrocardiogram (ECG) revealed significant ST-segment elevation in AVR and diffuse subendocardial ischemia, with ST elevation in lateral derivation and the right bundle branch block, as shown in Figure 1. The echocardiogram demonstrated anterior and anterolateral akinesia, hypokinesia in the remaining segments, and normal septal contractility, which resulted in a severe reduction in ejection fraction (EF 20%) and mild-to-moderate mitral regurgitation.

**Figure 2:**
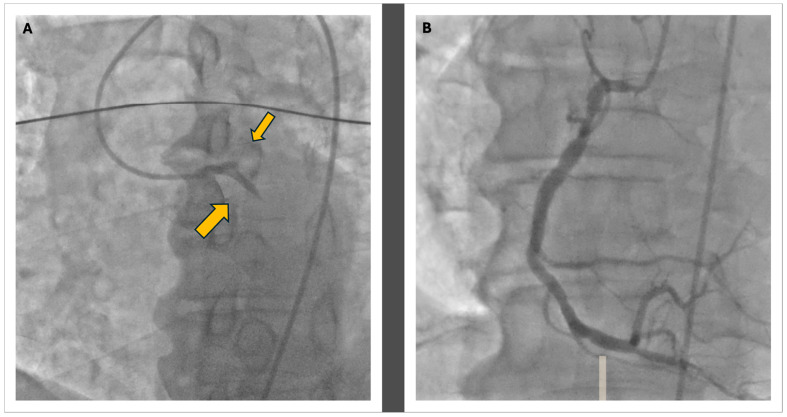
The coronary angiography: Panel (**A**) shows the double thrombosis of the left coronary artery at the proximal level of the LAD and the CFX with a TIMI 0 in both vessels, as the indicated by the yellow arrows; in Panel (**B**), the morphology of the right coronary artery is visible, displaying widespread atheromatous wall changes.

In response to the cardiogenic shock, which led to severe hypotension, inotropic therapy with norepinephrine and dobutamine was initiated. The patient was then transferred to the catheterization lab within 90 min of arrival to the emergency department, where acute subocclusion of the proximal circumflex branch and proximal LAD was identified as shown in Figure 2. Mechanical thrombus aspiration was performed through dual femoral access; the first was to perform angioplasty with greater support and the second was to position the intra-aortic balloon pump (IAPB) to support hemodynamics. Stents were placed in the proximal circumflex branch and proximal LAD. The distal LAD was treated with plain old balloon angioplasty (POBA). After the coronary angioplasty procedure, the patient was placed on dual antiplatelet therapy (DAPT) with acetylsalicylic acid 100 mg daily, following a loading dose of 250 mg IV, and prasugrel 10 mg daily after a loading dose of 60 mg orally.

**Figure 3:**
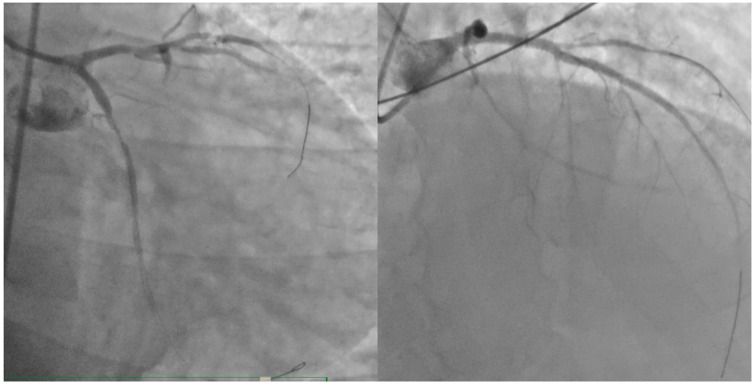
The final results (TIMI II flow) of percutaneous coronary intervention on left anterior discending artery and circumflex artery.

The final result was not optimal, but the vessels were open, as demonstrated in Figure 3. The peak troponin value was 180,900 ng/L, measured the following day (reference range: 2.5–53 ng/L). Plasma creatinine was 0.9 mg/dL (reference range up to 1.2 mg/dL), electrolytes were within normal limits, and total/LDL cholesterol levels were 200/160 mg/dL.

The patient was subsequently admitted to the intensive coronary care unit (CCU), where his stay was complicated by several issues. Initially, he underwent oro-tracheal intubation and, subsequently, due to difficulties in weaning from intubation, given the severe pulmonary history, the patient underwent a tracheostomy.

**Figure 4:**
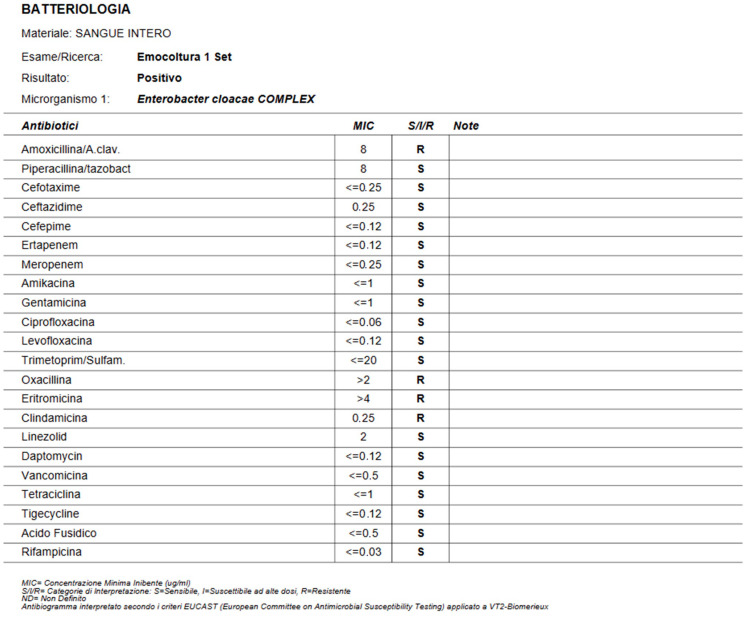
antibiogram showing the sensibility of Enterobacter Cloacae to meronemen.

Initially, he developed infections caused by various pathogens, leading to impaired gas exchange and necessitating orotracheal intubation. The pathogens were isolated from bronchoalveolar lavage the initial blood cultures were positive for *Staphylococcus capitis*, followed by *Staphylococcus hominis*, and were treated with vancomycin. Positive urine cultures for *Candida glabrata*, *Candida albicans*, and *Candida tropicalis* were not treated because it was considered colonization by the infectious disease specialist and because the patient was asymptomatic. Oral candidiasis was treated with Mycostatin. Following the resolution of these infections, the patient again presented with a febrile condition, elevated inflammatory markers, and positive blood cultures for *Enterobacter cloacae*, which was effectively treated with meropenem according to the antibiogram, as shown in Figure 4. Additionally, the patient developed severe bilateral pleural effusion, which required bilateral evacuative thoracentesis with drainage of serous fluid. He also experienced paralytic ileus, managed conservatively with the placement of a double-lumen nasogastric tube and parenteral nutrition. The patient’s nearly two-month stay in the CCU was marked by a challenging weaning process from orotracheal intubation and vasopressor support.

Moreover, during the intensive care stay, approximately 15 days after admission, the patient developed tetraparesis. A brain CT scan showed no signs of acute ischemic or hemorrhagic events, and electromyography revealed acute sensory-motor axonal polyneuropathy. During the hospitalization, this condition was managed with physiotherapy and corticosteroids, resulting in only a mild residual strength deficit in the left lower limb by the end of the treatment period.

Once the infectious issues were resolved, we attempted to strengthen our patient’s heart by administering two cycles of levosimendan, approximately 25 days apart, using a low infusion dose due to marked hypotension. This approach was chosen because, according to serial echocardiograms, the systolic function was not improving, with an EF around 20%, and particularly because of the difficult weaning from inotropic support.

Despite rapid coronary revascularization, ongoing vasopressor support, and meticulous vessel care, the patient’s cardiac function did not improve, and due to refractory heart failure leading to multiple pleural effusions, the patient was transferred to another center for evaluation of left ventricular assist device (LVAD) implantation after 2 months in our CCU. After the evaluation, the patient underwent LVAD evaluation.

## 3. Discussion

Myocardial infarction is the result of ischemic necrosis of cardiomyocytes downstream of coronary artery obstruction. This obstruction can be either subocclusive or occlusive, leading to either transmural necrosis, known as STEMI, or affecting only the endocardial portion, resulting in a non-ST elevation myocardial infarction (NSTEMI).

Typically, the obstruction is caused by thrombosis over an atherosclerotic plaque and, in the vast majority of cases, affects a single vessel at a time. The advent of coronary angioplasty has dramatically changed the course of this condition. By restoring coronary blood flow in the affected artery within a short time frame, it is possible to limit ischemia–reperfusion injury, significantly impacting patient mortality and prognosis. Additionally, when only one vessel is involved, the ischemic and potentially necrotic area will be more confined, although it may still be extensive.

The condition of a double culprit lesion leading to STEMI is an exceedingly rare scenario. However, a previous systematic review suggests that it may be significantly underreported due to its poor clinical outcome [1]. Its incidence is estimated to be at 2.5% [2] among patients undergoing PCI and about. This poor outcome is often attributed to cardiogenic shock and extensive myocardial ischemia in such cases, to the point of referring to this condition as the ‘Deadly double infarct syndrome’.

Several mechanisms could lead to this unusual occurrence:

1. diffuse atherosclerotic plaque instability: the simultaneous rupture or erosion of multiple atherosclerotic plaques can lead to thrombus formation in more than one coronary artery.
2. multiple coronary embolisms: embolic events can cause occlusion in multiple coronary vessels simultaneously.
3. diffuse coronary vasospasm: in rare cases, severe coronary vasospasm may affect multiple vessels simultaneously, leading to ischemia [3].
4. multiple in-stent thrombosis: in patients who have undergone previous PCI, multiple in-stent thromboses may result in simultaneous occlusion of two or more vessels.

According to Pollak’s data, males were significantly more affected by this rare condition, with smoking identified as the primary risk factor. The most common pattern of simultaneous occlusion involved the right coronary artery (RCA) and the circumflex artery (CFx), followed by occlusion of the right coronary artery and the left anterior descending artery. The least frequent scenario was the simultaneous occlusion of the two branches of the left coronary artery [2].

This condition, being very rare, also presents a challenge in determining the appropriate management approach. Hage [4], in a previous case report, emphasized the importance of intracoronary imaging to guide treatment; his case involved two culprit lesions, in both the RCA and LAD. After a failed thrombus aspiration on the LAD, they opted for a conservative approach, performing only an OCT 48 h later. This imaging confirmed the presence of eroded plaques with small adherent thrombi, without significant stenosis. However, their case involved a hemodynamically stable patient, whereas our patient was in cardiogenic shock, and we needed to restore intracoronary flow as quickly as possible.

In the case reported by Jariwala, the dual thrombosis involved the proximal LAD and the distal RCA. In this scenario, the left ventricle retained some revascularization from the circumflex branches, and although the right coronary artery had a distal thrombosis, the patient still presented in cardiogenic shock with a high-grade atrioventricular block that necessitated the placement of an intra-aortic balloon pump [5] and PCI.

In the case described by Kuzemczak, similar to our reported case, the patient exhibited dual thrombosis in the LAD and the circumflex arteries, with a TIMI 0 flow observed in the proximal LAD and at the mid-left circumflex artery, distally beyond the emergence of the first marginal branch. The patient experienced distress and tachycardia but was not in cardiogenic shock. Following PCI of both vessels without mechanical support, the patient was discharged without any adverse events after a week-long hospitalization [6].

In the case reported by Fukaya, the dual thrombosis involved the proximal LAD with a TIMI 0 flow and the mid-distal circumflex artery. The patient was hemodynamically stable at presentation but became unstable during PCI and went into cardiogenic shock, necessitating the use of an intra-aortic balloon pump for circulatory support, similar to our case. The PCI was successfully completed by stenting both vessels. We do not have discharge data for this patient, but it is known that at the time of publication, the patient had a biplane ejection fraction of 60% and had not experienced any major events [7]. The previously published case reports are summarized in Table 1.

Compared to previously reported cases, our patient presented in critical condition from the onset; he was in cardiogenic shock and significantly hypotensive. Additionally, our patient developed a newly diagnosed right bundle branch block (RBBB). According to data from a meta-analysis, new-onset RBBB in patients with myocardial infarction is an unfavorable prognostic factor, as it is associated with a higher risk of long-term mortality and cardiogenic shock [8].

Moreover, compared with the two discussed previous patients, our patient, to our knowledge, was the first reported case that had a TIMI 0 flow on both the proximal LAD immediately after the left main trunk and on the proximal circumflex before the emergence of any marginal branch. This scenario was equivalent to a TIMI 0 flow at the level of the left main trunk; indeed, the ECG was quite typical of a left main trunk involvement with strongly positive AVR, despite the presence of a right bundle branch block.

In line with what is known in the literature, our patient also has a significant smoking history, which may suggest that smoking could play a role in the pathogenesis of this rare occurrence. However, our patient developed one of the rarest combinations, presenting with thrombosis of both the circumflex artery and the LAD.

This case report also provides a point of reflection on the approach to patients with STEMI ACS in the context of multivessel coronary artery disease.

The 2023 acute coronary syndrome (ACS) guidelines provide recommendations for revascularization in the case of non-culprit vessels. In hemodynamically stable STEMI cases, there is a Class I recommendation to revascularize the non-culprit either during the index procedure or within 45 days. However, in cases of cardiogenic shock, the Class I recommendation is to revascularize only the culprit lesion [9].

Therefore, the guidelines lack explicit recommendations for managing cases with two different culprit lesions. In this case, we can assume that the primary culprit was the CX coronary artery lesion, which may have triggered the release of pro-inflammatory cytokines and vasoconstrictive molecules. These factors likely contributed to the destabilization of a pre-existing plaque in the left LAD.

Nevertheless, in emergency situations, it is very challenging to determine which of the two vessels is the initial culprit lesion that led to low cardiac output and, subsequently, caused acute thrombosis of the second culprit lesion; it may be reasonable to treat both culprit lesions if the patient stabilizes.

Moreover, the ACS 2023 guidelines recommend avoiding invasive procedures if the patient’s symptoms have started more than 48 h prior, and if the patient is asymptomatic [9]. In our case, the patient’s symptoms began two days before presentation, aligning with the 48 h guideline threshold. However, given that the patient was still symptomatic upon arrival at the emergency department and presented in cardiogenic shock in critical condition and life-threatening, we opted to proceed with PCI. During the procedure, we decided to revascularize both the circumflex artery and the LAD, prioritizing the patient’s critical condition and the need for prompt intervention given that both the anterior septum and the posterolateral wall were not yet akinetic.

Self-critique. Our patient was transferred to a secondary care facility for evaluation for LVAD implantation, intended as a bridge to heart transplantation. Despite all efforts, including two months of hospitalization in the coronary intensive care unit after a double cycle of levosimendan, there was no improvement in left ventricular function. This lack of improvement could be attributed, at least in part, to certain decisions made during the treatment course. Notably, heart failure therapy was not fully optimized due to the patient’s unstable hemodynamic status; for example, beta-blocker therapy was maintained at very low doses following a brief episode of complete atrioventricular block. Implanting an ICD at that time might have allowed for more aggressive beta-blocker titration, but given the patient’s critical condition, we believe it was not the best option. Furthermore, after this short episode of total AV block, which lasted only a few minutes, the patient did not experience any recurrence. We initiated an aldosterone antagonist and valsartan with the aim of eventually introducing sacubitril/valsartan. However, this could not be achieved due to the patient’s persistent hypotension, which prevented the transition to this combination therapy.

Retrospectively, there was a discussion within our team regarding the possibility of using an alternative circulatory support system. There is a lack of substantial evidence regarding which circulatory support device is the most effective on survival rate. On the one hand, in the IABP-SHOCK II trial, the routine use of IABP in patients with ACS and cardiogenic shock did not reduce 30-day, 1-year, or 6-year mortality [10]. On the other hand, the randomized trials ISAR-SHOCK [11] and IMPRESS [12] compared the use of IABP and Impella (specifically Impella 2.5 and CP) in patients with acute myocardial infarction complicated by cardiogenic shock, demonstrating the absence of statistically significant differences in mortality between the two groups (at 30 days and at 6 months, respectively), albeit at the cost of a higher rate of bleeding in patients treated with Impella. The timing for the implantation of the support device differed between the two studies. In ISAR-SHOCK, the device was implanted following coronary revascularization, while in IMPRESS, the decision regarding when to implant the device was left to the operator’s judgment, allowing for implantation before, after, or during primary PCI. Recently, findings have indicated the benefits of the systematic and timely implantation of the Impella device in patients experiencing acute myocardial infarction with cardiogenic shock, implemented before revascularization procedures and prior to administering inotropes and vasopressors [13]. However, in the case of our patient, with the entire left coronary artery occluded, there was no possibility of waiting to position the Impella before reopening the vessel because the patient was unstable, and it was necessary to act quickly; therefore, we preferred to initiate positive inotropic therapy, reopen the two occluded vessels, and subsequently opted to place the intra-aortic balloon pump.

**Table 1:** The lists of published cases, with the mode of presentation, the treatment, and the outcome.

Author and Year	Type of ACS	Presentation	Outcome	Vessels	Treatment
Mahorkar AV et al., 2023 [14]	Infero-posterior	Severe hypokinesia, ejection fraction 30%, symptoms of infero-posterior wall MI	Discharged on day 5 in stable condition	Distal RCA, LCx	Drug-eluting stents, PTCA for RCA and LCx
Kanei et al., 2009 [15]	Inferior STEMI	Cardiogenic shock		RCA, LAD	Intra-aortic balloon pump, thrombectomy, PCI with DES to RCA and LCx
Fukaya et al., 2011 [7]	Anterolateral STEMI	Haemodinamically stable, FE 60%	No data	LAD, LCx	Intra-aortic balloon pump, PCI with DES
Kuzemczak et al., 2018 [6]	STEMI due to simultaneous occlusion of LAD and LCx, rapid clinical course	Haemodynamically stable, but very sofferent	Discharged in stable condition	LAD, LCx	PCI with DES
Pankaj Jariwala et al., 2023 [5]	Inferior STEMI	Cardiogenic shock with 2:1 atrio-ventricular block		Proximal LAD and RCA	IAPB, PCI with DES
Hage et Al., 2021 [4]	Anterior and inferior STEMI	Haemodinamically stable	Discharge in stable conditions	Proximal LAD and mid RCA	Failed PCI in LAD and medical treatment in RCA
Marchi ed Al. 2024 [16]	Inferior STEMI and Wellens type A	Hypotensive	Discharge in stable conditions	Proximal RCA and mid LAD	PCI

## 4. Conclusions

This rare case of simultaneous thrombosis in two coronary arteries leading to STEMI and cardiogenic shock underscores the complexities of management in such patients. Further research is needed to optimize revascularization strategies and improve outcomes, highlighting the importance of a tailored and multidisciplinary approach in critical cases.

## Data Availability

The original contributions presented in this study are included in the article. Further inquiries can be directed to the corresponding author.
